# A novel defined programmed cell death related gene signature for predicting the prognosis of serous ovarian cancer

**DOI:** 10.1186/s13048-024-01419-y

**Published:** 2024-04-29

**Authors:** Feng Zhan, Yina Guo, Lidan He

**Affiliations:** 1 College of Engineering, Fujian Jiangxia University, Fuzhou, Fujian 350108 China; 2 School of Electronic Information Engineering, Taiyuan University of Science and Technology, Taiyuan, Shanxi 030024 China; 3 Department of Obstetrics and Gynecology, the First Affiliated Hospital of Fujian Medical University, Fuzhou, Fujian 350004 China

**Keywords:** Ovarian cancer, Serous ovarian cancer, Overall survival, Clinical prediction, Precision medicine, Single-cell RNA sequencing

## Abstract

**Purpose:**

This study aims to explore the contribution of differentially expressed programmed cell death genes (DEPCDGs) to the heterogeneity of serous ovarian cancer (SOC) through single-cell RNA sequencing (scRNA-seq) and assess their potential as predictors for clinical prognosis.

**Methods:**

SOC scRNA-seq data were extracted from the Gene Expression Omnibus database, and the principal component analysis was used for cell clustering. Bulk RNA-seq data were employed to analyze SOC-associated immune cell subsets key genes. CIBERSORT and single-sample gene set enrichment analysis (ssGSEA) were utilized to calculate immune cell scores. Prognostic models and nomograms were developed through univariate and multivariate Cox analyses.

**Results:**

Our analysis revealed that 48 DEPCDGs are significantly correlated with apoptotic signaling and oxidative stress pathways and identified seven key DEPCDGs (CASP3, GADD45B, GNA15, GZMB, IL1B, ISG20, and RHOB) through survival analysis. Furthermore, eight distinct cell subtypes were characterized using scRNA-seq. It was found that G protein subunit alpha 15 (GNA15) exhibited low expression across these subtypes and a strong association with immune cells. Based on the DEGs identified by the GNA15 high- and low-expression groups, a prognostic model comprising eight genes with significant prognostic value was constructed, effectively predicting patient overall survival. Additionally, a nomogram incorporating the RS signature, age, grade, and stage was developed and validated using two large SOC datasets.

**Conclusion:**

GNA15 emerged as an independent and excellent prognostic marker for SOC patients. This study provides valuable insights into the prognostic potential of DEPCDGs in SOC, presenting new avenues for personalized treatment strategies.

**Supplementary Information:**

The online version contains supplementary material available at 10.1186/s13048-024-01419-y.

## Introduction

Ovarian cancer (OC) is a prevalent gynecologic malignancy that affects the female reproductive system. Serous ovarian cancer (SOC) is the most common subtype, accounting for approximately 70–80% of all cases OC. SOC is characterized by high mortality and the rising number of cases each year, posing a significant threat to women’s well-being [1]. Despite significant advancements in debulking surgery and chemotherapy treatments, the overall survival rate of SOC remains suboptimal, with only approximately 30% of patients surviving beyond five years [2]. The tumor heterogeneity of SOC patients presents a significant challenge for predicting overall survival and treatment efficacy. Traditional prognostic indicators have included pathological types and stages, the presence of residual disease after debulking surgery, serum markers like CA125 and HE4, and imaging indicators such as ultrasound [3]. However, these predictors no longer meet the clinical requirements of precision medicine in managing SOC. Therefore, it is imperative to expedite the development of efficacious prognostic markers and novel treatment targets to enhance the survival rates of SOC.

Programmed cell death (PCD) is a genetically regulated process of cellular demise that serves a vital function in maintaining homeostasis [4]. Extensive research has focused on PCD in malignancies, revealing its significance in the development and dissemination of malignant cells [5]. Studies have shown that PCD, such as ferroptosis, necroptosis, and pyroptosis, are closely associated with OC’s occurrence, progression, and therapeutic potential [6]. However, intra-tumoral heterogeneity remains a significant challenge in the context of ovarian cancer [7, 8], with implications for cancer progression and survival rates [9, 10]. Therefore, investigating how PCD contributes to the heterogeneity of SOC is essential for providing precise treatment guidance and improving overall survival rates.

The advent of single-cell RNA sequencing (scRNA-seq) has revolutionized the study of tumoral heterogeneity in OC. It has facilitated the identification of critical factors and cellular subpopulations involved in tumor progression [11–13]. By enhancing our understanding of tumoral heterogeneity, scRNA-seq offers novel perspectives on cancer biology [14]. Liu et al. utilized scRNA-seq to identify four M2 tumor-associated macrophage (TAM)-associated genes that possess predictive significance in OC patients [15]. Similarly, Tan et al. utilized scRNA-seq to reveal dynamic alterations occurring in the immunological milieu of bladder cancer and establish a predictive model [16]. Moreover, scRNA-seq has led to the discovery of new malignant cell populations associated with unfavorable prognostic outcomes in OC [17]. Additionally, Yu et al. [18] identified flavin-containing monooxygenase 2 as a novel cancer-associated fibroblast-derived biomarker for predicting the course of OC. However, despite these advancements, a comprehensive study of the relationship between PCD and tumor heterogeneity in SOC still needs to be conducted. The detailed mechanism of PCD in SOC’s heterogeneity remains thinly investigated.

In this study, we identified 48 differentially expressed programmed cell death-related genes (DEPCDGs) associated with apoptotic signaling and oxidative stress pathways. We further identified seven key DEPCDGs (CASP3, GADD45B, GNA15, GZMB, IL1B, ISG20, and RHOB) with prognostic significance through survival analysis. We identified eight distinct cell subtypes corresponding to 13 clusters using scRNA-seq on SOC tumor tissue samples. Interestingly, G protein subunit alpha 15 (GNA15) exhibited low expression across these single-cell subtypes and was strongly associated with immune cells in the RNA-seq data. To further investigate GNA15, we conducted a single-gene bioinformatics analysis and constructed a prognostic model. This model displayed promising predictive ability in both the TCGA and GEO cohorts, establishing GNA15 as a valuable autonomous prognostic determinant for SOC patients. Overall, our scRNA-seq investigation offers crucial insights into the complex tumoral heterogeneity of SOC, shedding light on potential avenues for developing novel therapeutic strategies.

## Materials and methods

### Data preparation

We collected 375 tumor tissues samples (TCGA-OV) (Homo sapiens) from the University of California Santa Cruz Xena (UCSC Xena, https://xenabrowser.net/datapages/) [19], ensuring all selected samples had complete survival data. These samples provided transcriptome sequencing data with fragments per kilobase million (FPKM) expression values, along with relevant clinical information, such as age, histologic grade, and clinical stage (Table S1). Additionally, we sourced 88 healthy ovarian tissue samples (Homo sapiens) from the Genotype-Tissue Expression database (GTEx, https://gtexportal.org/home/) [20]. These samples provided the transcriptome sequencing FPKM expression profile and count matrix. In addition, we downloaded the RNA-seq dataset GSE63885 [21] from the Gene Expression Omnibus database(GEO, https://www.ncbi.nlm.nih.gov/geo/) through the “GEOquery” R package [22]. This dataset was derived from the GPL570 [HG-U133_Plus_2] Affymetrix Human Genome U133 Plus 2.0 Array, which focuses on Homo sapiens and comprises a total of 75 ovary tumor samples after removing missing survival data. We further downloaded scRNA-seq data GSE184880 from the GEO database [23], which was derived from the platform GPL24676 Illumina NovaSeq 6000 (Homo sapiens) and contained seven SOC samples without treatment and five control samples. The inclusion and exclusion criteria of this study were defined as follows: (1) The inclusion criteria: ① Patients diagnosed and treated for SOC initially, excluding those with recurrent SOC; ② Complete clinical and pathological data. (2) The exclusion criteria: ① Excluded patients with incomplete pathologica or clinical data; ② Patients with incomplete follow-up time, other causes of death and unknown death status; ③ Patients with multiple tumors and non-primary tumors.

### Identification and enrichment analysis of DEPCDGs

Initially, we collected 268 programmed cell death genes (PCD genes) from existing literature sources [24, 25]. Using scRNA-seq datasets, we identified 3,000 cell Differential genes (cellDiffgenes) intersecting with PCD genes to obtain our target gene. Subsequently, we obtained a combined dataset by emerging OC samples from TCGA databases with healthy ovarian samples from GTEx databases using the “ComBat” R package [26]. We successfully identified differentially expressed programmed cell death genes (DEPCDGs) in SOC by analyzing these target genes in the combined dataset using the “limma” R package [27] (*p* < 0.05 and | logFC (Fold Change) |> 1). Among the DEPCDGs, those with a *p*-value less than 0.05 and logFC more than 1 were classified as up-regulated. Conversely, DEPCDGs with a *p*-value less than 0.05 and logFC less than -1 were categorized as down-regulated. The “heatmap” and “ggplot2” R packages were employed to generate visual representations of heat and volcano maps.

Gene Ontology (GO) [28] is a commonly used approach in conducting comprehensive investigations of functional enrichment studies. This method encompasses the examination of cell composition (CC), biological process (BP), and molecular function (MF). Similarly, the Kyoto Encyclopedia of Genes and Genomes (KEGG) [29] is an extensively utilized database encompassing comprehensive data on genomes, biological processes, diseases, and pharmaceuticals. To analyze the functional characteristics and pathway enrichment of the DEPCDGs, we performed GO and KEGG analyses using the “ClusterProfler” R package [30] and graphically represented using the “ggplot2” R package. For statistical significance, we defined enrichment as a function or pathway term with a false discovery rate (FDR) less than 0.25 and a *p*-value less than 0.05. The *p*-value adjustment used the Benjamini-Hochberg (BH) approach [31].

### Identification of key DEPCDGs based on survival analysis

We performed survival analysis on DEPCDGs utilizing the “survival” R package [32] and identified key prognostic genes with statistical significance (*p* < 0.05). These key prognostic genes were then selected as key DEPCDGs for further analysis.

### Expression of key DEPCDGs on scRNA-seq data

We imported raw data from SOC samples in the scRNA-seq dataset utilizing the “Seurat” R package (version 4.0) [33] and created Seurat objects for subsequent analysis. We applied gene < 200 or > 3,000 filtration conditions to remove low-quality cells [34]. The proportion of mitochondrial genes in relation to the total genetic material can indicate cellular homeostasis. Cells with mitochondrial gene content > 10% were excluded from further analysis due to potential stress. Consequently, we obtained a final set of 3,555 cells for subsequent analysis.

The scRNA-seq data was normalized using the LogNormalize method. We identified cellDiffgenes in individual cells after controlling for the relationship between average expression and dispersion. Next, we employed Principal Component Analysis (PCA) to decentralize all genes and cluster all cells. Subsequently, we displayed the resulting cell subclusters utilizing Uniform Manifold Approximation and Projection (UMAP) [35]. The cell type of each cluster was determined by referencing the Human Primary Cell Atlas (HPCA) dataset using the singleR method [36].

### Evaluation of immune cell infiltration

CIBERSORT (https://cibersortx.stanford.edu/) is a computational tool that employs linear support vector regression to deconvolute the transcriptome expression matrix. Its purpose is to estimate the composition and number of immune cells within a mixture of cells [37]. We utilized the CIBERSORT algorithm to determine the fraction of 22 immune cell types, exploring the association between key DEPCDGs and the immunological microenvironment. The relative abundance of immune cells in a dataset sample can be calculated using single-sample gene set enrichment analysis (ssGSEA) [38]. The immune cell enrichment scores of combined datasets were assessed by using ssGSEA with the “GSVA” R package. This analysis was performed based on the relative abundance of each immunocyte infiltrate in every sample. Samples with a *p*-value less than 0.05 were filtered and included in the output. Finally, the correlation analysis results between key DEPCDGs and infiltrating immune cells in combined datasets were visually represented using the “pheatmap” R package. The core genes for further analysis were selected based on the most relevant key DEPCDGs.

### Difference and enrichment analysis of core gene

Within the TCGA-OV dataset, SOC patients were classified into high- and low-expression groups by utilizing the median value of the core gene. Differential analysis was performed using the “limma” R package to identify the differentially expressed genes (DEGs) with statistical significance (*p* < 0.05 and | logFC |> 1). To determine the biologically significant pathways mediated by the hub gene, we performed GO and KEGG enrichment analyses using the “ClusterProfiler” R package [39] and visualized using the “ggplot2” R package.

In order to evaluate the contribution of DEGs to the phenotype, we employed Gene Set Enrichment Analysis (GSEA) [40]. GSEA is a computational methodology in which genes in a predetermined genetic set are analyzed within the gene list ordered by phenotypic correlation. We performed enrichment analysis on all DEGs with high and low phenotype correlations in both groups using the “clusterProfiler” R package. The parameters employed for GSEA were as follows: a seed value of 2020 was utilized for random number generation, 10,000 computations were performed, and each gene set contained a minimum of 10 genes and a maximum of 500 genes. Enrichment analysis was conducted using the “c2.cp.v7.2.symbols.gmt” gene set obtained from the Molecular Signatures Database (MSigDB) [41] via the GSEA method. We defined statistically significant enrichment as a pathway or function term with an FDR less than 0.25 and a *p*-value less than 0.05. The *p*-value correction was conducted using the BH method.

### Construction and evaluation of a prognosis model based on core gene

DEGs identified from hub gene grouping were selected as candidate genes. To investigate their prognostic value for SOC, we assess the correlation between these candidate genes and survival outcomes via univariate Cox regression analysis using “survival” and “forestplot” R packages. Based on the DEGs with noteworthy prognostic value (*p* < 0.05), we conducted multivariate Cox regression analysis to calculate regression coefficients and develop a risk model. This model enabled us to assign a risk score (RS) to each tumor sample using the following formula,

1 $$RS=\sum_{k=1}^N\limits\left(coef\left(k\right)+x(k)\right),$$

Where *N* denotes the number of genes, *coef*(*k*) represents the multivariate Cox regression coefficient, and *x*(*k*) represents the expression value of each gene.

Our data analysis identified seven prognosis-related feature genes: CD3E, CD2, IL2RG, FCGBP, RARRES1, UBD, VSIG4, and STAB1. Subsequently, the patients were classified into high- and low-risk groups according to the median of the RS. To evaluate the predictive effectiveness of our risk model, we employed several methods: the Risk Triptych, time-dependent receiver operator characteristic (time-ROC) curve analysis [42], Kaplan–Meier (K-M) curve analysis [43], and decision curve analysis (DCA).

### Construction and evaluation of a nomogram based on the risk score

In order to determine the potential independence of the prognostic factor, we assess the correlation between survival outcomes and variables such as RS, age, stage, and grade via univariate Cox regression using the “survival” and “forestplot” R packages. Furthermore, we explored independent influencing factors through multivariate Cox regression and visualized them in forest plots. A nomogram was ultimately constructed utilizing the RS and clinical characteristics in order to forecast the prognosis of SOC. The performance of this nomogram was subsequently assessed through the use of the Calibration curve and ROC curve.

### Statistical analysis

We performed statistical analysis in this study using RStudio (version 4.2). The Kruskal–Wallis test was employed to compare groups consisting of three or more, while the Wilcoxon rank sum test was utilized for the comparison of two groups. Spearman’s method was employed for correlation analysis. The “survival” R package was employed to conduct univariate and multivariate Cox analyses. Additionally, survival differences were displayed using K-M survival curves. The Log-rank test was employed to evaluate the extent of the disparity in survival durations among the various groups of patients. All statistical tests were conducted with bilateral *p*-values, and a significance level of *p* < 0.05 was employed.

## Results

### Workflow chart

**Fig. 1:**
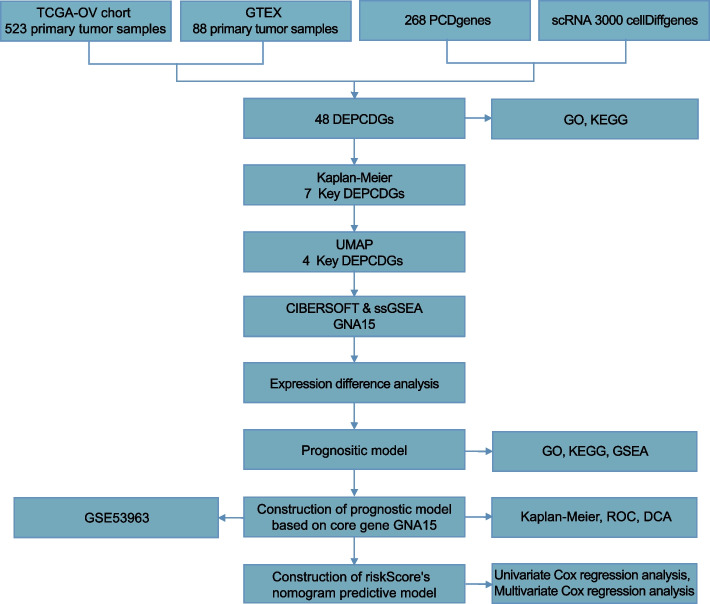
Flow chart for the comprehensive analysis of DEPCDGs. DEPCDGs, differentially expressed programmed cell death genes

In order to provide a clearer understanding of the research process, we presented the workflow of our study in Fig. 1.

### Identification and pathway enrichment analysis of DEPCDGs

**Fig. 2:**
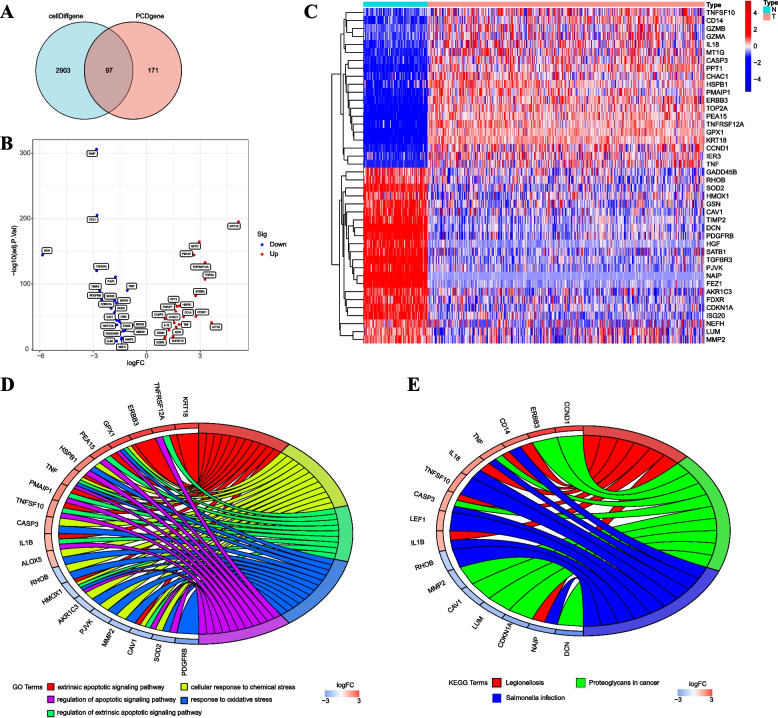
Identification and pathway enrichment analysis of DEPCDGs. **A** Venn diagram displaying the overlap genes between cell Diff genes and PCD genes (3,000 PCD genes shown in red, 268 cellDiffgenes shown in blue, and 97 PCD-related cellDiffgenes overlap between both sets). **B** The volcano plot of DEPCDGs in combined datasets. **C** Clustered heatmap of DEPCDGs in combined datasets. **D** GO enrichment analysis of DEPCDGs (FDR < 0.05). **E** KEGG enrichment analysis of DEPCDGs (FDR < 0.05). Blue represents the normal group; orange represents the tumor group. DEPCDGs, differentially expressed programmed cell death genes; PCD, programmed cell death; GO, Gene Ontology; KEGG, Kyoto Encyclopedia of Genes and Genomes; FDR, false discovery rate

We identified a total of 97 PCD-related cellDiffgenes by intersecting 3,000 cellDiffgenes from the scRNA-seq dataset with 268 PCD genes from existing literature sources. Moreover, the 171 genes are uniquely associated with the PCD gene set, which were not present among the cellDiffgenes., hinting at their potential involvement in specific PCD pathways relevant to ovarian cancer (Fig. 2A). A combined dataset was obtained by emerging OC samples from TCGA databases with healthy ovarian samples from GTEx databases. We identified 48 DEPCDGs by performing differential analysis on the expression of PCD-related cellDiffgenes in the combined dataset using the “limma” R package ( |logFC|> 1 and *p* < 0.05). Among them, 18 genes were up-regulated ( logFC > 1 and *p* < 0.05), and 30 genes were down-regulated ( logFC < -1 and *p* < 0.05). The volcano plot visualized these DEPCDGs (Fig. 2). The differential expression of DEPCDGs between various sample groups in combined datasets was analyzed. The results of this analysis were visualized using a heatmap plot generated by the “pheatmap” R package (Fig. 2C).

Forty-eight DEPCDGs were analyzed by GO and KEGG pathway enrichment analysis (Table 1). GO analysis revealed enrichment of DEPCDGs in pathways such as extrinsic apoptotic signaling pathway, cellular response to chemical stress, regulation of extrinsic apoptotic signaling pathway, response to oxidative stress, and regulation of apoptotic signaling pathway (Fig. 2D). In the KEGG pathway analysis, several enriched pathways of DEPCDGs were identified, including legionellosis, proteoglycans in cancer, and salmonella infection (Fig. 2E).

**Table 1:** Results of GO and KEGG enrichment analysis for DEPCDGs

ONTOLOGY	ID	Description	GeneRatio	BgRatio	*p*	p.adjust	qvalue	Count
BP	GO:0097191	extrinsic apoptotic signaling pathway	11/48	221/18800	7.12E-12	1.71E-08	9.44E-09	11
BP	GO:0062197	cellular response to chemical stress	12/48	332/18800	2.97E-11	3.57E-08	1.97E-08	12
BP	GO:2001236	regulation of extrinsic apoptotic signaling pathway	9/48	153/18800	1.58E-10	1.27E-07	6.98E-08	9
BP	GO:0006979	response to oxidative stress	12/48	433/18800	6.30E-10	3.78E-07	2.09E-07	12
CC	GO:0043202	lysosomal lumen	4/48	97/19594	9.29E-05	0.007156323	0.005711273	4
CC	GO:0045121	membrane raft	6/48	326/19594	0.000137999	0.007156323	0.005711273	6
CC	GO:0098857	membrane microdomain	6/48	327/19594	0.00014032	0.007156323	0.005711273	6
CC	GO:0005775	vacuolar lumen	4/48	174/19594	0.000861927	0.032968701	0.02631145	4
MF	GO:0005126	cytokine receptor binding	6/47	272/18410	6.36E-05	0.009836915	0.007129429	6
MF	GO:0030291	protein serine/threonine kinase inhibitor activity	3/47	33/18410	8.06E-05	0.009836915	0.007129429	3
MF	GO:0019887	protein kinase regulator activity	5/47	207/18410	0.000178926	0.012633891	0.009156573	5
MF	GO:0032813	tumor necrosis factor receptor superfamily binding	3/47	49/18410	0.000264592	0.012633891	0.009156573	3
KEGG	hsa05134	Legionellosis	6/39	57/8163	2.42E-07	3.03E-05	1.63E-05	6
KEGG	hsa05205	Proteoglycans in cancer	9/39	205/8163	3.68E-07	3.03E-05	1.63E-05	9
KEGG	hsa05132	Salmonella infection	9/39	249/8163	1.88E-06	0.000103558	5.55E-05	9
KEGG	hsa05210	Colorectal cancer	6/39	86/8163	2.83E-06	0.000116793	6.26E-05	6

### Identification of key DEPCDGs based on survival analysis

**Fig. 3:**
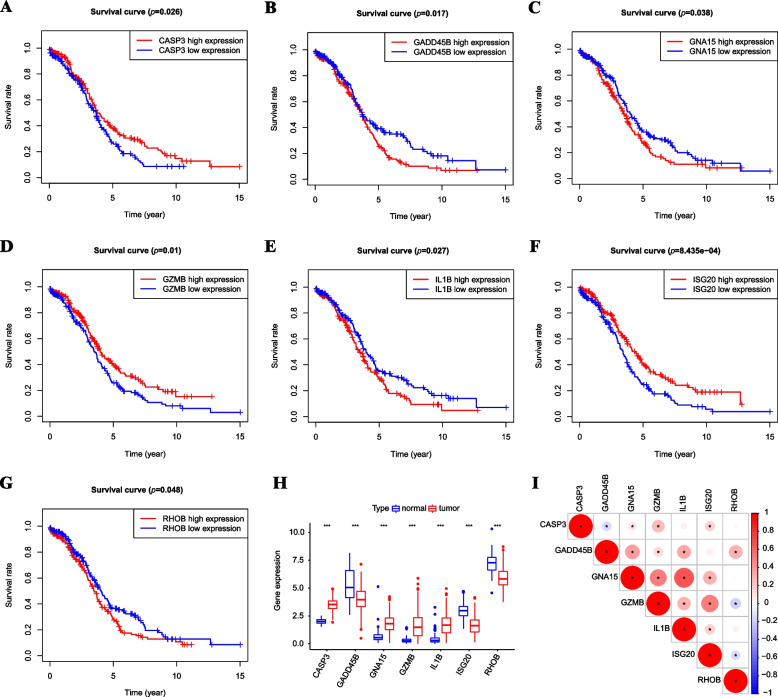
Identification of key DEPCDGs based on survival analysis. **A-G** Kaplan–Meier survival curves of seven key DEPCDGs in TCGA-OV: CASP3 (**A**), GADD45B (**B**), GNA15 (**C**), GZMB (**D**), IL1B (**E**), ISG20 (**F**), RHOB (**G**). **H** Group comparison boxplot of seven key DEPCDGs. **I** Correlational analysis of seven key DEPCDGs. Red represents positive correlation, blue represents negative correlation. *, *p* < 0.05; **, *p* < 0.01; ***, *p* < 0.001; DEPCDGs, differentially expressed programmed cell death genes; TCGA-OV, The Cancer Genome Atlas—Ovarian Cancer; CASP3, the third comparative assessment of techniques of protein structure prediction; GADD45B, growth arrest and DNA-damage-inducible protein 45 beta; GNA15, G protein subunit alpha 15; GZMB, Granzyme B, IL1B, cytokine interleukin-1β; ISG20, Interferon-stimulated gene 20; RHOB, RhoB

We conducted survival analysis on 48 DEPCDGs in the TCGA-OV group and identified seven key DEPCDGs with prognostic significance in SOC (*p* < 0.05). These genes were the third comparative assessment of techniques of protein structure prediction (CASP3), growth arrest and DNA-damage-inducible protein 45 beta (GADD45B), GNA15, Granzyme B (GZMB), cytokine interleukin-1β (IL1B), Interferon-stimulated gene 20 (ISG20), and RhoB (RHOB) (*p* < 0.05) (Fig. 3A-G). The differential expression of these seven genes in the combined dataset shown in Fig. 3H, with GASP3, GNA15, GZMB, and IL1B strongly expressed in the tumor group, while GADD45B, ISG20, and RHOB were lowly expressed (*p* < 0.001). Additionally, correlation analysis revealed that GNA15 and IL1B were relatively highly correlated (Fig. 3I). These findings suggest the potential prognostic significance of these key DEPCDGs in SOC.

### Expression of key DEPCDGs on scRNA-seq data

In our study, RNA sequencing was performed on single cells from seven ovarian cancer samples. To ensure the overall quality of single-cell data, we implemented filtering conditions to eliminate low-quality cells and batch effects. Specifically, we set the filtering condition as follows: the number of RNA features (nFeature_RNA) had to be between 200 and 3,000, and the percentage of mitochondrial genes (percent.mito) had to be below 20%. Through this filtering process, we successfully identified and retained a total of 3,555 high-quality cells.

**Fig. 4:**
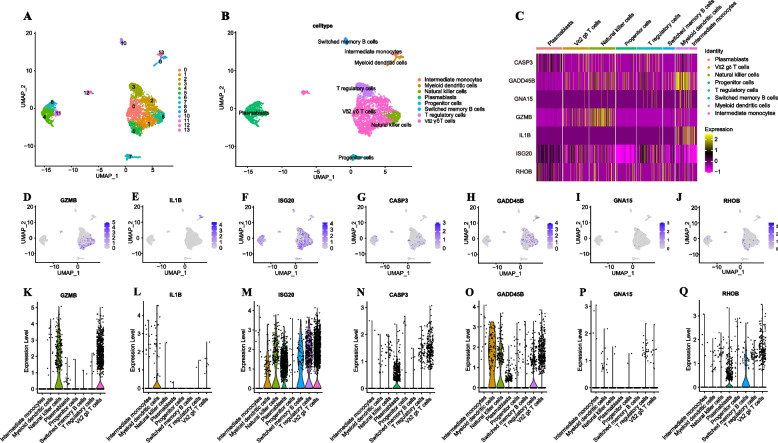
Expression of key DEPCDGs on scRNA-seq data. **A** UMAP plot of 13 cell clusters with similar gene expression profiles. **B** UMAP plot of eight cell subtypes. **C** Heatmap of key DEPCDGs’ expression level in eight cell subtypes. **D-J** UMAP plots of key DEPCDGs’ expression level in eight cell subtypes. GZMB (**D**), IL1B (**E**), ISG20 (**F**), CASP3 (**G**), GADD45B (**H**), GNA15 (**I**), RHOB (**J**). **K-Q** Violin diagrams of key DEPCDGs’ expression level in eight cell subtypes. GZMB (**K**), IL1B (**L**), ISG20 (**M**), CASP3 (**N**), GADD45B (**O**), GNA15 (**P**), RHOB (**Q**). Red represents low expression; yellow represents high expression. DEPCDGs, differentially expressed programmed cell death genes; UMAP, uniform manifold approximation and projection; CASP3, the third comparative assessment of techniques of protein structure prediction; GADD45B, growth arrest and DNA-damage-inducible protein 45 beta; GNA15, G protein subunit alpha 15; GZMB, Granzyme B, IL1B, cytokine interleukin-1β; ISG20, Interferon-stimulated gene 20; RHOB, RhoB

Following data normalization and gene centralization, we performed PCA dimensionality reduction by extracting the top 3, 000 cellDiffgenes at the single-cell level. To identify distinct groups of cells with similar gene expression profiles, we employed the top 50 principal components for clustering. This clustering analysis yielded 13 independent clusters, which were subsequently visualized using UMAP (Fig. 4A).

Using the HPCA data to identify cell type of each cluster, we found eight cell subtypes after annotating, including intermediate monocytes, myeloid dendritic cells (mDCs), NK cells, plasmablasts, progenitor cells, switched memory B cells, Tregs, and Vdelta2 gamma-delta (Vδ2 gδ) T cells (Fig. 4B). Expression levels of seven key DEPCDGs in various cell types visually showed by the UMAP (Fig. 4D-J). Our findings revealed that GADD45B exhibited a high expression level in intermediate monocytes, GZMB was highly expressed in NK cells, and IL1B showed significant expression in intermediate monocytes. Interestingly, GNA15 displayed low expression across all cell subtypes. These results were further validated by the violin diagram (Fig. 4K-Q) and heatmap (Fig. 4C).

### Immune cell infiltration in the transcriptome and its correlation with key DEPCDGs

**Fig. 5:**
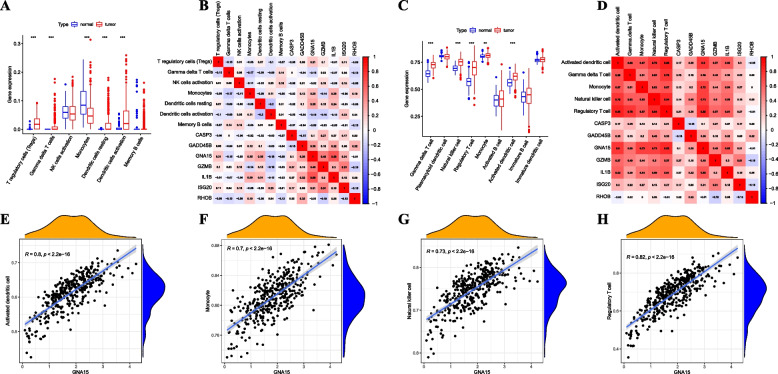
Immune infiltration analysis in combined datasets. **A** Group comparison chart of immune cell infiltration analysis in combined datasets by the CIBERSOFT method. **B** Heatmap of the relationship between key DEPCDGs and specific immune cell subtypes calculated by the CIBERSOFT method. **C** Group comparison chart of immune cell infiltration analysis in combined datasets by the ssGSEA method. **D** Heatmap of the relationship between key DEPCDGs and specific immune cell subtypes by the ssGSEA method. **E–H** Correlation analysis between GNA15 and specific immune cell subtypes (activated dendritic cell (**E**), monocyte (**F**), NK cell (**G**), Tregs (**H**)). Red represents the tumor group; blue represents the normal group. *, *p* < 0.05; **, *p* < 0.01; ***, *p* < 0.001; DEPCDGs, differentially expressed programmed cell death genes; ssGSEA, single-sample gene set enrichment analysis; GNA15, G protein subunit alpha 15

In order to examine the relationship between key DEPCDGs and immune infiltration in the transcriptome, we conducted an analysis of immune cell infiltrates in the combined datasets utilizing CIBERSOFT and ssGSEA methods. The CIBERSORT algorithm analyzed immune infiltrates abundance of Tregs, gδT cells, NK cells activated, monocytes, dendritic cells resting, dendritic cells activated, and B cells memory. The results revealed that the tumor group exhibited higher immune infiltration levels of Tregs, gδT cells, dendritic cells resting, and dendritic cells activated compared to the normal group. Additionally, the infiltration abundance of monocytes displayed a statistically significant decrease in the tumor group (Fig. 5A, *p* < 0.001). Using the ssGSEA algorithm, we analyzed immune infiltrates’ abundance of gδT cells, plasmacytoid dendritic cells, NK cells, Tregs, monocytes, activated B cells, activated dendritic cells, immature B cells, and immature dendritic cells. The results indicated that gδT cells, NK cells, Tregs, monocytes, and activated dendritic cells exhibited higher immune infiltration levels in the tumor group compared to the normal group (Fig. 5C, *p* < 0.01). We screened out GNA15 as a core DEPCDG in SOC, as it showed a strong correlation with dendritic cells resting in the CIBERSORT algorithm (*R* = 0.35), none of the other genes were highly correlated with cells (Fig. 5B). Similarly, GNA15 demonstrated a high correlation with all identified immune cell subtypes in the ssGSEA algorithm(all *R* ≥ 0.58) (Fig. 5D). Specific high correlation results were shown in Fig. 5E-H, where GNA15 exhibited a correlation with activated dendritic cells (*R* = 0.80, *p* < 0.001, Fig. 5E), monocytes (*R* = 0.70, *p* < 0.001, Fig. 5F), NK cells (*R* = 0.73, *p* < 0.001, Fig. 5G), and Tregs (*R* = 0.82, *p* < 0.001, Fig. 5H).

### Analysis of variance and functional enrichment based on GNA15

**Fig. 6:**
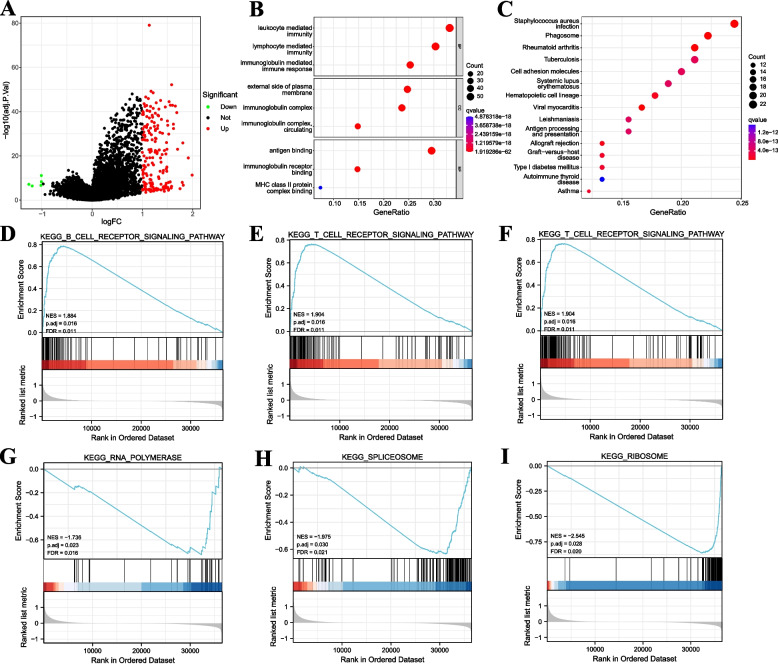
Single-gene bioinformatic analysis of GNA15. **A** Volcano plot of DEGs in high and low GNA15 expression groups in TCGA-OV. **B** GO enrichment analysis of DEGs n high and low GNA15 expression groups in TCGA-OV. **c** KEGG enrichment analysis of DEGs n high and low GNA15 expression groups in TCGA-OV. **D-F** GSEA analysis of high GNA15 expression group. B cell receptor signal transduction (**D**), T cell receptor signaling pathway (**E**), TOLL-like receptor signaling pathway (**F**). **G-I** GSEA analysis of low GNA15 expression group. RNA polymerase (**G**), spliceosome (**H**), ribosome (**I**). GNA15, G protein subunit alpha 15; DEGs, differentially expressed genes; TCGA-OV, The Cancer Genome Atlas—Ovarian Cancer; KEGG, Kyoto Encyclopedia of Genes and Genomes; GSEA, gene set enrichment analysis

Based on our analysis results, we specifically focused on the core gene GNA15 for single-gene bioinformatics analysis. The HGSOC patients were categorized into high- and low-expression groups using the median value of the GNA15 in the TCGA-OV dataset. Through differential analysis, we identified 5 down-regulated DEGs and 180 up-regulated DEGs (|logFC|> 1, *p* < 0.05) (Fig. 6A). To gain insights into biological pathways modulated by GNA15, we conducted GO and KEGG enrichment analyses on these DEGs. The GO enrichment analysis revealed a predominant involvement of DEGs in leukocyte-mediated immunity, B cell-mediated immunity, antigen-binding pathways, and other relevant pathways (Fig. 6B). Meanwhile, the KEGG enrichment analysis discovered that DEGs enrichment in pathways associated with *staphylococcus aureus* infection, phagosome, and other related pathways (Fig. 6C). Additionally, we employed GSEA analysis to investigate the implications of GNA15 expression further (Table 2). The results of our study indicate a significant association between the high expression of GNA15 and B cell receptor signaling pathway, T cell receptor signaling pathway, and Toll-like receptor signaling pathway (Fig. 6D-F). Conversely, low expression of GNA15 was enriched in the ribosome, spliceosome, and RNA polymerase pathways (Fig. 6G-I).

**Table 2:** TOP 20 results of GSEA for TCGA-OA dataset

ID	setSize	enrichmentScore	NES	*p*	p.adjust	qvalues
KEGG_RIBOSOME	88	-0.881642594	-2.35478993	1.00E-10	1.42E-09	8.42E-10
KEGG_CYTOKINE_CYTOKINE_RECEPTOR_INTERACTION	263	0.818850512	2.211493886	1.00E-10	1.42E-09	8.42E-10
KEGG_CHEMOKINE_SIGNALING_PATHWAY	188	0.829317495	2.189465877	1.00E-10	1.42E-09	8.42E-10
KEGG_TOLL_LIKE_RECEPTOR_SIGNALING_PATHWAY	102	0.870671429	2.177440979	1.00E-10	1.42E-09	8.42E-10
KEGG_NATURAL_KILLER_CELL_MEDIATED_CYTOTOXICITY	132	0.836256916	2.152736693	1.00E-10	1.42E-09	8.42E-10
KEGG_ANTIGEN_PROCESSING_AND_PRESENTATION	81	0.886790285	2.144698591	1.00E-10	1.42E-09	8.42E-10
KEGG_AUTOIMMUNE_THYROID_DISEASE	50	0.945297962	2.141011487	1.00E-10	1.42E-09	8.42E-10
KEGG_HEMATOPOIETIC_CELL_LINEAGE	85	0.875785337	2.13708209	1.00E-10	1.42E-09	8.42E-10
KEGG_LEISHMANIA_INFECTION	69	0.884430569	2.104761525	1.00E-10	1.42E-09	8.42E-10
KEGG_CELL_ADHESION_MOLECULES_CAMS	131	0.809666125	2.084812817	1.00E-10	1.42E-09	8.42E-10
KEGG_VIRAL_MYOCARDITIS	68	0.876151547	2.079476127	1.00E-10	1.42E-09	8.42E-10
KEGG_LYSOSOME	121	0.802078295	2.045087934	1.00E-10	1.42E-09	8.42E-10
KEGG_FOCAL_ADHESION	199	0.759222161	2.007323323	1.00E-10	1.42E-09	8.42E-10
KEGG_SYSTEMIC_LUPUS_ERYTHEMATOSUS	56	0.900458059	2.087142871	1.35E-10	1.78E-09	1.05E-09
KEGG_COMPLEMENT_AND_COAGULATION_CASCADES	69	0.866012639	2.060930668	3.31E-10	3.83E-09	2.27E-09
KEGG_ALLOGRAFT_REJECTION	35	0.946485716	2.018567298	3.26E-10	3.83E-09	2.27E-09
KEGG_T_CELL_RECEPTOR_SIGNALING_PATHWAY	108	0.801131037	2.015904273	1.40E-09	1.52E-08	9.00E-09
KEGG_JAK_STAT_SIGNALING_PATHWAY	155	0.744247373	1.932325326	1.62E-09	1.66E-08	9.84E-09
KEGG_INTESTINAL_IMMUNE_NETWORK_FOR_IGA_PRODUCTION	45	0.914558119	2.039161727	2.73E-09	2.66E-08	1.57E-08
KEGG_TYPE_I_DIABETES_MELLITUS	41	0.917534976	2.019273297	4.09E-09	3.60E-08	2.13E-08

### Construction and evaluation of a prognostic model based on GNA15

**Fig. 7:**
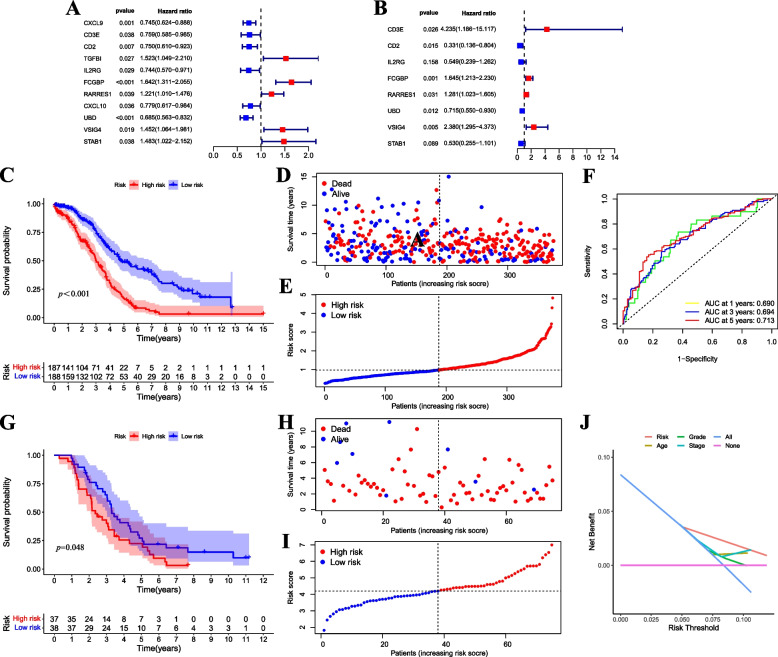
Construction evaluation of a predictive model based on GNA15. **A** Univariate Cox regression analysis of DEGs in the TCGA-OV dataset. **B** Multivariate Cox regression analysis of DEGs in the TCGA-OV dataset. **C** The K-M survival curve analysis of prognostic models in the TCGA-OV dataset (*p* < 0.001). **D** Distribution of SOC patients with different RS in the TCGA-OV dataset. **E** Survival status analysis of SOC patients with different RS in TCGA-OV dataset. **F** timeROC analysis of 1-, 3- and 5-year in the TCGA-OV dataset. **G** The K-M survival curve analysis of the prognostic model in the GSE63885 dataset (*p* < 0.05). **H** Distribution of patients with various RS in the GSE63885 dataset. **I** Survival status analysis of patients with various RS in the GSE63885 dataset. **J** DCA curve of the RS’ prediction power in the TCGA-OV dataset. Red represents the high-risk group; blue represents the low-risk group. GNA15, G protein subunit alpha 15; DEGs, differentially expressed genes; TCGA-OV, The Cancer Genome Atlas—Ovarian Cancer; SOC, serous ovarian cancer; K-M, Kaplan–Meier; ROC, receiver operator characteristic; DCA, decision curve analysis

A predictive model was created utilizing the core gene GNA15. Initially, a univariate Cox regression analysis was performed on DEGs between high and low GNA15 expression. Our results revealed 11 genes with significant prognostic value (*p* < 0.05) in SOC (Fig. 7A). Subsequently, we performed multivariate Cox regression analysis on these 11 genes to construct the predictive model consisting of eight genes: CD3E, CD2, IL2RG, FCGBP, RARRES1, UBD, VSIG4, and STAB1 (Fig. 7B). We categorized patients into high- and low-risk groups based on the RS median. The Risk Triptych showed the strong predictive capacity of the model in both TCGA-OV and GSE63885 datasets (Fig. 7D-E, H-I). Furthermore, the K-M survival curve indicated that the high-risk group had worse prognoses compared to the low-risk group in both TCGA-OV and GSE63885 datasets (*p* < 0.0001, *p* = 0.048) (Fig. 7C, G). The timeROC curve showed the RS’s strong predictive ability for overall survival (OS) in SOC patients, with AUCs of 0.690, 0.694, and 0.713 for 1-year, 3-year, and 5-year respectively (Fig. 7F). Finally, the DCA confirmed the substantial predictive ability of the RS signature (Fig. 7J).

### Construction of a nomogram prediction model based on RS

**Fig. 8:**
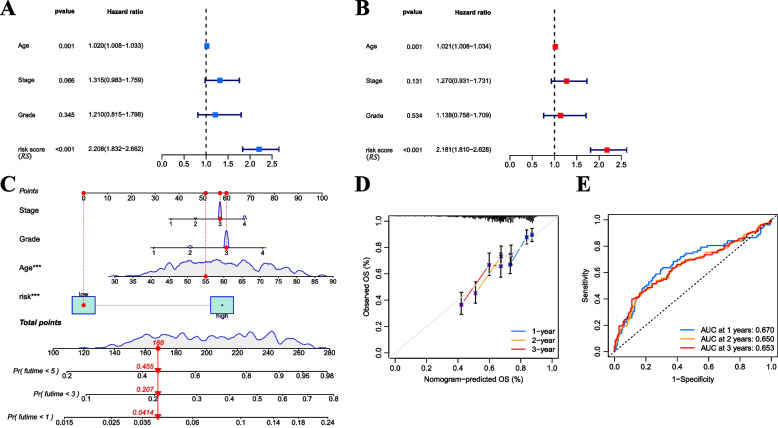
Construction of a nomogram prediction model based on RS. **A** Univariate Cox regression analysis of RS and clinical features in the TCGA-OV dataset. **B** Multivariate Cox regression analysis of RS and clinical features in the TCGA-OV dataset. **C** Nomogram prediction model included stage, grade, age, and RS. **D** Calibration curve of the nomogram’s prognostic prediction. **E** ROC curve of the nomogram’s prognostic prediction. RS, risk score; TCGA-OV, The Cancer Genome Atlas—Ovarian Cancer; ROC, receiver operator characteristic

We conducted a comprehensive analysis to determine whether RS could used as an independent prognostic factor. Firstly, a univariate Cox regression analysis was performed to assess the relationship between RS, age, stage, grade, and OS. The forest plots revealed that age and RS are significantly related to OS (Fig. 8A). Further analysis was undertaken using multivariate Cox regression, considering the aforementioned variables. Remarkably, both RS and age emerged as independent prognostic factors for predicting patients’ OS without relying on other clinical features (Fig. 8B). Subsequently, we constructed a nomogram model that incorporates RS along with three other clinical features for forecasting SOC patient outcomes (Fig. 8C). The good predictive power of this model was demonstrated by the Calibration curve (Fig. 8D). Moreover, the ROC curve analysis of the nomogram displayed its precise predictive ability for OS of SOC patients, with AUC values of 0.670, 0.650, and 0.653 for the 1-year, 3-year, and 5-year OS predictions, respectively (Fig. 8E).

## Discussion

SOC is an aggressive neoplasm of the reproductive system. Despite improvements in therapy, the high intra-tumor heterogeneity makes improving the overall survival rate challenging. scRNA-Seq technologies have been widely recognized for their ability to examine tumor heterogeneity through the evaluation of gene expression at the individual cell level [44]. Several studies have focused on developing accurate and sensitive predictive models for SOC prognosis, incorporating immune genes, serum biomarkers, and other factors [45–47]. However, to enhance the validity and reliability of these models, it is imperative to take into account the heterogeneity of tumor samples. PCD is a fundamental process for cellular self-repair and regulation, and its dysregulation contributes to malignant tumor development and metastasis [5]. PCD-related genes are critical in SOC [48]. In this study, we employed a combination of scRNA-Seq and bulk RNA-Seq techniques to examine tumor heterogeneity and investigate the involvement of PCD in the progression of SOC. To the best of our understanding, this bioinformatics analysis is the initial demonstration of the role of PCD and tumor heterogeneity on the prognosis of SOC using scRNA-Seq, and we establish prognostic signatures based on core DEPCDGs.

Our study identified 48 DEPCDGs contributing to the heterogeneity of SOC by performing differential analysis using TCGA-OV and GTEx datasets. Through GO analysis, we determined enrichment pathways for these DEPCDGs, including the extrinsic apoptotic signaling pathway, cellular response to chemical stress, regulation of the extrinsic apoptotic signaling pathway, response to oxidative stress, and regulation of the apoptotic signaling pathway. Additionally, the KEGG analysis revealed enrichment pathways, such as legionellosis, proteoglycans in cancer, and salmonella infection. In small intestinal neuroendocrine neoplasia, GNA15 inhibits cell proliferation and promotes apoptosis through the NFκB and Akt signaling pathways [49]. LINC02474 inhibits apoptosis by impeding GZMB expression in colorectal cancer [50]. Moreover, proteoglycans have been found to play a significant role in cancer progression by influencing cancer cell aggressiveness, angiogenesis, and stromal microenvironment [51]. These studies provide support for the validity of the current study.

Among the genes studied, GNA15 exhibited consistently low expression across all eight cell subtypes and strongly correlated with immune cell subtypes. GNA15 is a member of the GNA gene family, which is crucial in regulating cell proliferation and apoptosis. It is expressed in highly specific cell types, such as hematopoietic [52] and epithelial cells [53], during certain stages of differentiation. GNA15 has been identified as highly expressed in small intestinal neuroendocrine neoplasia [49] and pancreatic ductal adenocarcinoma [54], correlating with poor survival. It is worth noting that prior research has yet to explore the specific role of GNA15 in the SOC tumorigenesis and progression mechanism. We predict that GNA15 is involved in the development and advancement of SOC, serving as a potential theoretical foundation for SOC treatment and prognosis.

The comparative analysis conducted on groups exhibiting contrasting levels of GNA15 expression demonstrated that a total of 180 genes showed up-regulation, whereas a mere 5 genes displayed down-regulation. We investigated the role of GNA15 in various biological pathways through GO and KEGG analyses. GO analysis revealed the involvement of GNA15 in leukocyte and B cell-mediated immunity, as well as antigen-binding pathways. Meanwhile, KEGG analysis identified *staphylococcus aureus* infection, phagosome, and other biological processes. These findings underscore the diverse functions of GNA15 in various cellular pathways. Furthermore, GSEA analysis demonstrated a significant correlation between high expression of GNA15 and the activation of T-cell receptor and B-cell receptor signaling pathways. In contrast, low expression is correlated with ribosome and spliceosome pathways, which indicates that GNA15 is engaged in the regulation of cellular processes associated with immunological signaling and protein synthesis. Moreover, a study by Zeng et al. [55] highlighted that the carcinogenic role of miR-211-5p mediated by GNA15, which modifies the immune function of the tumor microenvironment extrinsically while also impacting the intracellular processes of pyroptosis and glycolysis in melanoma cells. Additionally, the expression levels of GNA15 have been implicated in the effectiveness of anti-tumor chemotherapeutic medicines [56]. Overall, these findings underscore the multifaceted functions of GNA15 in tumor cellular processes. Further investigation into the role of GNA15 in these pathways can enhance our understanding of cellular mechanisms and contribute to the development of novel treatments.

To evaluate the predictive capability of GNA15, we constructed a prognostic model incorporating eight genes (CD3E, CD2, IL2RG, FCGBP, RARRES1, UBD, VSIG4, and STAB1), which were identified as DEGs in the GNA15 high- and low-expression groups. This model demonstrated predictive solid ability in the TCGA-OV and GSE63885 datasets, confirming that the resulting RS signature can be an independent prognostic factor for SOC. These findings suggest that GNA15 holds promising potential in forecasting overall survival in SOC patients, indicating its crucial role in PCD for heterogeneity of SOC. For instance, Innamorati et al. [54] conducted pancreatic ductal adenocarcinoma (PDAC), Zanini et al. [49] focused on small intestinal neuroendocrine neoplasia, and Li et al. [57] investigated acute myeloid leukemia. These studies provide valuable insights into the role of GNA15 in identifying and predicting the progression and prognosis of these malignancies. These studies align with our findings and further support the idea that GNA15 is involved in diverse malignancies. The relationship between increased expression of GNA15, early relapse, and poor survival in SOC may be attributed to the induction of a stem cell-like phenotype in human ovarian cancer cells through the downregulation of AKT activity. Additionally, GNA15 facilitates cellular signaling and migratory properties in transformed cells. Moreover, high expression of GNA15 is linked to the heterogeneity and prognosis of SOC. These findings suggest that GNA15 holds promise in predictive and prognostic analyses of SOC.

Despite offering valuable insights, this study has several limitations that warrant attention. Firstly, the exclusion criteria applied to patient selection enhance the quality and integrity of the data, thereby stabilizing and consistent results while ensuring the accuracy, reliability, and repeatability of the findings. However, this approach may also introduce sample selection bias, potentially limiting the generalizability of our conclusions. Secondly, our study does not statistically compare the clinical efficacy of our nomogram with any previously developed and validated models. This comparison is crucial for establishing the relative performance and potential advantages of our approach. Thirdly, the data analyzed in this study were exclusively derived from public databases, including TCGA, GTEx, and GEO, without incorporating raw data from our own investigations. This reliance on secondary data sources may affect the direct applicability of our findings to other datasets or clinical scenarios.

## Conclusion

In conclusion, our research demonstrates the potent prognostic value of GNA15 for the overall survival of SOC patients. We have developed a novel single-cell prognostic model for SOC, shedding new light on the progression of this disease. Our study highlights the critical role of GNA15 in predictive analysis. Performing an in-depth analysis of gene patterns in SOC can further enhance our understanding of the disease’s etiology, prognosis, and treatment options. Future research in this area should focus on investigating the potential implications of GNA15 and related genes in the context of SOC. By doing so, we can make significant strides in advancing our knowledge of this complex disease and potentially identifying new therapeutic targets. In conclusion, we emphasize the significance of investigating the processes of carcinogenesis using the methodology of single-cell genomics, as it has the potential to yield vital insights into the underlying mechanisms of SOC formation.

### Supplementary Information

**Supplementary Material 1.**

## Data Availability

No datasets were generated or analysed during the current study.
