# Supplementary material for: A novel defined programmed cell death related gene signature for predicting the prognosis of serous ovarian cancer
Source: J Ovarian Res. 2024 Apr 29;17:92. doi: 10.1186/s13048-024-01419-y (PMC11057167; doi:10.1186/s13048-024-01419-y)
Supplement: Supplementary file 1 — Supplementary Material 1. [file 13048_2024_1419_MOESM1_ESM.doc]

**Table S1** Baseline data for SOC patients (TCGA-OV). SOC, serous ovarian cancer; TCGA-OV, The Cancer Genome Atlas - Ovarian Cancer.

| **Characteristics** | **Overall** |
| --- | --- |
| Age, n (%) |  |
| <= 60 | 206 (55%) |
| > 60 | 169 (45%) |
| Histologic grade, n (%) |  |
| G1 | 1 (0.3%) |
| G2 | 42 (11.4%) |
| G3 | 321 (88%) |
| G4 | 1 (0.3%) |
| Clinical stage, n (%) |  |
| Stage I | 1 (0.3%) |
| Stage II | 22 (5.9%) |
| Stage III | 294 (78.4%) |
| Stage IV | 58 (15.5%) |
